# Comparison of the diversity of cultured and total bacterial communities in marine sediment using culture-dependent and sequencing methods

**DOI:** 10.7717/peerj.10060

**Published:** 2020-10-21

**Authors:** Meng Wang, Samina Noor, Ran Huan, Congling Liu, JiaYi Li, Qingxin Shi, Yan-Jiao Zhang, Cuiling Wu, Hailun He

**Affiliations:** 1 School of Life Science, Central South University, Changsha, China; 2 Qingdao Agricultural University, Qingdao, China; 3 Changzhi Medical College, Changzhi, China

**Keywords:** Marine sediment, Culturable microbial diversity, Microbial diversity, Media components, Physiochemical parameters

## Abstract

Despite recent great advances in microbial culture, most microbes have not yet been cultured, and the impact of medium composition on the isolation of microbes from natural systems has not been elucidated. To optimize media for culturing marine microbes, microbial communities in three sediment samples were described using high-throughput sequencing (HTS) and culture-dependent techniques. HTS revealed communities dominated by *Gammaproteobacteria*, and culture-based methods revealed communities dominated by *Actinobacteria*. Among the total operational taxonomic units (OTUs) from the HTS dataset, 6% were recovered in the culture collection. Four potentially novel bacterial strains belonging to *Oceaniovalibus*, *Psychrobacter* and *Salegentibacter* were isolated. The combination of media cultured more taxa than any single medium. Nutrient-rich and single-carbon/nitrogen-source media supported the growth of relatively few taxa, and the quality of nitrogen strongly influenced the types of bacteria isolated.

## Introduction

Microbes in natural environments play important roles in global carbon and nitrogen cycles (Louca, Parfrey & Doebeli, 2016). Although considerable progress has recently been achieved in microbial culture, a high proportion of microbes remain uncultured in most biomes (Lloyd et al., 2018; Steen et al., 2019). Many phylogenetically novel groups have never been cultured, and their physiologies have therefore only been inferred from genomics and environmental characteristics (Stewart, 2012; Lloyd et al., 2018). With the development of sequencing technologies, investigators can try to reveal potential novel functions and phylogenetic relationships by sequencing the genomes of entire communities to circumvent the limitation and laboriousness of isolation work (Ronaghi, Uhlén & Nyrén, 1998; Rinke et al., 2013). Nonetheless, isolation of microbes is still necessary for the exploitation of bioactive compounds (Ling et al., 2015), descriptions of new taxa of prokaryotes, and experimental validation of microbial, ecological, and evolutionary processes.

Many strategies such as high-throughput culturing, physically separating species-specific growth enhancing compounds, and simulating the natural environment have been used to increase the number of microbial species cultured from environmental samples (Kaeberlein, Lewis & Epstein, 2002; Henson et al., 2016; Strandwitz et al., 2019). Medium composition plays an important role as particular substrates can select for particular microbes. For example, salicylic acid selects for *Flavobacterium* sp. 40 and *Tetracoccus* sp. 273 (Lebeis et al., 2015), and the abundant marine bacterium *Candidatus* Pelagibacter ubique requires reduced sulphur compounds (Tripp et al., 2008). Toerien (1967) also found that the morphology of culturable microbes also depended on the substrate used. However, systematic research on differences in culturable microbes in different media is still lacking. To improve the efficiency of microbial culture, the effects of medium components on microbial culture require further study.

In this study, three sediment samples were collected from the South China Sea, and six different media were used to isolate their microbes. The microbial diversity of the marine sediment samples was assessed by high-throughput sequencing (HTS) in parallel. The feasibility of combining various media to improve the diversity of culturable microbial taxa detected was systematically evaluated. The physical and chemical parameters of the media were further analyzed, and the relationships between medium components and culturable microbial communities were initially revealed. We found that low-nutrient and multiple-carbon/nitrogen-source media worked best.

## Materials & Methods

### Marine sediment collection

Three marine sediment samples (C1, I1, and X4) were collected from the South China Sea in June 2014 (Table S1). These samples were packed into sterile centrifuge tubes, transported to the laboratory and stored at 4 °C for later use.

### DNA extraction and 16S rRNA gene fragment sequencing

DNA was extracted with an E.Z.N.A.^®^ Soil DNA Kit according to the supplier’s instructions (Omega Bio-Tek, USA). The DNA concentration was measured with a NanoDrop-200 microspectrophotometer (Allsheng, China). The 16S rRNA gene was amplified using the barcoded primers 515F (5′-GWA TTA CCG CGG CKG CTG-3′) and 806R (5′-CCG TCA ATT CMT TTR AGT TT-3′) (Bergmann et al., 2011). A negative control (water instead of DNA) was included in the PCR, and no bands were observed in the negative control. PCR products were sent to BGI Tech Co. Ltd. (Wuhan, China) for further purification and HTS using the HiSeq PE250 system.

### Isolation and characterization of culturable bacterial strains

Culturable bacteria were isolated by six different media: Emerson agar (EM), Zobell 2216E (2216E), R2A agar (R2A), modified yeast mannitol agar medium (MCCM), modified chemically defined medium (CDM), and mineral basal medium (MBM) (Table 1). A 5.0-g sediment sample was dissolved in 20 ml of sterilized artificial seawater and placed in a conical flask in a shaker (30 min, 180 rpm). Then, the mixture was incubated until the two phases separated. The supernatants were serially diluted with sterilized artificial seawater, and 100 µl diluents were spread onto plates. The plates were incubated at 16 ° C until stable colonies formed. Each gradient was performed in triplicate. Negative controls included sterilized artificial seawater, and no colonies were noted in the negative controls during the culture process. To count the number of strains, plates with 25–250 colonies were selected (Tomasiewicz et al., 1980). Colonies with different morphologies were subcultured into pure cultures by inoculating them into freshly prepared agar plates (Selvin et al., 2019). The number of similar colonies was recorded for subsequent identification and statistics. The total DNA of bacteria was extracted using a Biospin Bacteria Genomic DNA Extraction Kit (Bioer, China). The 16S rRNA gene was amplified using the primers 27F (5′-AGA GTT TGA TCC TGG CTC AG-3′) and 1492R (5′-CGG TTA CCT TGT TAC GAC TT-3′) (Weisburg et al., 1991). PCR products were detected on a 1.5% agarose gel and sequenced using the Sanger sequencing method (BGI Inc., Guangzhou, China).

**Table 1:** The components of EM, 2216E, R2A, MCCM, CDM and MBM media.

	Medium(g/L)	Reference
Emerson agar (EM)	glucose:10, yeast extract:10, beef extract:4, peptone:4, artificial seawater(ASW):1 L, 121 °C, 30 min	Shirling & Gottlieb (1966)
Zobell 2216E (2216E)	peptone:5, yeast extract:1, FePO_4_⋅ 4H_2_O:0.01, ASW:1 L, 121 °C, 30 min	ZoBell (1941)
R2A agar (R2A)	yeast extract:0.5, peptone:0.5, casein hydrolysate:0.5, glucose:0.5, soluble starch:0.5, sodium pyruvate:0.3, K_2_HPO_4_:0.3, MgSO_4_⋅ 7H_2_O:0.05, ASW:1 L, 115 °C, 15 min	*Massa et al. (1998)*
Modified yeast mannitol agar medium (MCCM)	KH_2_PO_4_:0.2, K_2_HPO_4_:0.8, MgSO_4_⋅ 7H_2_O:0.2, CaSO_4_⋅ 2H_2_O:0.1, Na_2_MoO_4_⋅ 2H_2_O:trace, yeast extract:0.5, mannitol:20, FeCl_3_:trace, ASW: 1 L, 121 °C, 30 min	DeLajudie et al. (1998)
Modified chemically defined medium (CDM)	Na_2_HPO_4_⋅ 7H_2_O:7.9, KH_2_PO_4_:1.5, NH_4_Cl:1, MgSO_4_⋅ 7H_2_O:0.3, KCl:0.5, CaCl_2_:0.025, sodium lactate:7 ml, microelement:5 ml, vitamin:100 µl, ASW: 1 L, 121 °C, 30 min	*Battaglia-Brunet et al. (2002)*
Mineral basal medium (MBM)	K_2_HPO_4_:0.5, NH_4_H_2_PO_4_:0.5, (NH_4_)_2_SO_4_:1, MgSO_4_:0.2, KNO_3_:3, FeSO_4_:0.05, ASW:1 L, 121 °C, 30 min	Pucci et al. (2012)
Artificial seawater (ASW)	NaCl:28.15, MgSO_4_⋅ 7H_2_O:6.92, KCl:0.67, MgCl_2_⋅ 6H_2_O:5.51, CaCl_2_⋅ H_2_O:1.45, ultrapure water:1 L, 121 °C, 30 min	

### Physicochemical analysis of media

The total organic carbon (TOC), total phosphorus (TP), organic nitrogen (ON) and inorganic nitrogen (IN) contents of the media were measured. All the liquid media were sterilized before physicochemical analysis. A TOC analyzer (TOC-5000A, Shimadzu, Japan) was used to determine TOC, and the media were diluted 1000 times with ultrapure water before testing. Total nitrogen (TN) was measured using an alkaline potassium persulfate oxidation-ultraviolet spectrometer (UV-2450, Shimadzu, Japan). Ammoniacal nitrogen (NH_4_^+^-N) was measured by spectrophotometry with salicylic acid. Nitrate nitrogen (NO_3_^−^-N) was measured using UV spectrophotometry without digestion (Bao, 2011). Nitrite nitrogen (NO_2_^−^-N) was measured using a spectrophotometer by a coupling reaction with naphthylenediamine hydrochloride. The concentration of IN was determined by the following equation: c(IN)=c(NH_4_^+^-N)+c(NO_3_^−^-N)+c(NO_2_^−^-N). The ON concentration was determined by c(ON)=c(TN)-c(IN) (Li et al., 2006; Ren et al., 2006). TP was determined by the molybdoantimonyl phosphoric acid photometric method (UV-2450, Shimadzu, Japan) using persulfate as the reducing agent (121 °C, 30 min) (Guo, 2009). The media listed above were diluted 20 times before testing.

### Bioinformatics and statistical analysis

For HTS data, sequencing reads were assigned to each subsample according to unique barcodes. Then, paired-end reads from the original DNA fragments were merged using Fast Length Adjustment of SHort reads (FLASH, v1.2.11) after removing the barcode and primer sequences (Magoč & Salzberg, 2011). QIIME and USEARCH (v7.0.1090) were used to filter the reads and to select the operational taxonomic units (OTUs) with 97% similarity, respectively (Edgar, 2013). Finally, the resulting OTUs_(97%)_ were aligned against the Silva (Silva_119_release_aligned) database. To evaluate microbial richness and diversity, the abundance-based coverage estimator (ACE), Chao1, Shannon and Simpson indices were calculated using the mothur program. A Venn diagram was constructed in R (v3.1.1) with the ”VennDiagram” package. To characterize similarities in microbial communities among different sediment samples, hierarchical cluster analysis at the genus level was performed by Past3 software (version 3.22) (Aguilar et al., 2016). To compare the recoveries of bacteria between culture-dependent and HTS methods, the sequences obtained from cultured strains and the HTS dataset were combined and clustered with 97% identity by the UPARSE algorithm (Lee et al., 2016). The 16S rRNA gene sequences were deposited in the NCBI Sequence Read Archive under accession number PRJNA577514.

For the 16S rRNA gene sequences, bacterial taxonomy was assigned at the OTUs_(98%)_ level using a 98% confidence threshold (Stackebrandt & Ebers, 2006). The EzBioCloud platform (https://www.ezbiocloud.net/identify) was employed to identify the most closely related taxon according to the 16S rRNA gene sequence (Kim et al., 2012). The linear discriminant analysis (LDA) effect size (LEfSe) approach was used to determine bacterial community differences and search for biomarkers in the media. The LEfSe approach was implemented by online tools with default settings (http://huttenhower.sph.harvard.edu/galaxy/) (Segata et al., 2011). All tests for significance were two sided, and *p*-values <0.05 were considered statistically significant. To determine the associations between culturable bacterial communities and medium components, redundancy analysis (RDA) was performed using CANOCO software (version 5.0). The relationships between the microbes and individual physicochemical factors were determined by correlation tests using Pearson correlation coefficient analysis software (version 3.6.1). To characterize similarities in the culturable bacterial communities between sediment samples, hierarchical cluster analysis was performed by Past3 software (version 3.22) (Aguilar et al., 2016). The 16S rRNA gene sequences of representative taxa for each sediment sample were deposited in the NCBI GenBank database with accession numbers MK841046 –MK841311.

## Results

### Sediment microbial communities detected by the HTS method

**Figure 1:**
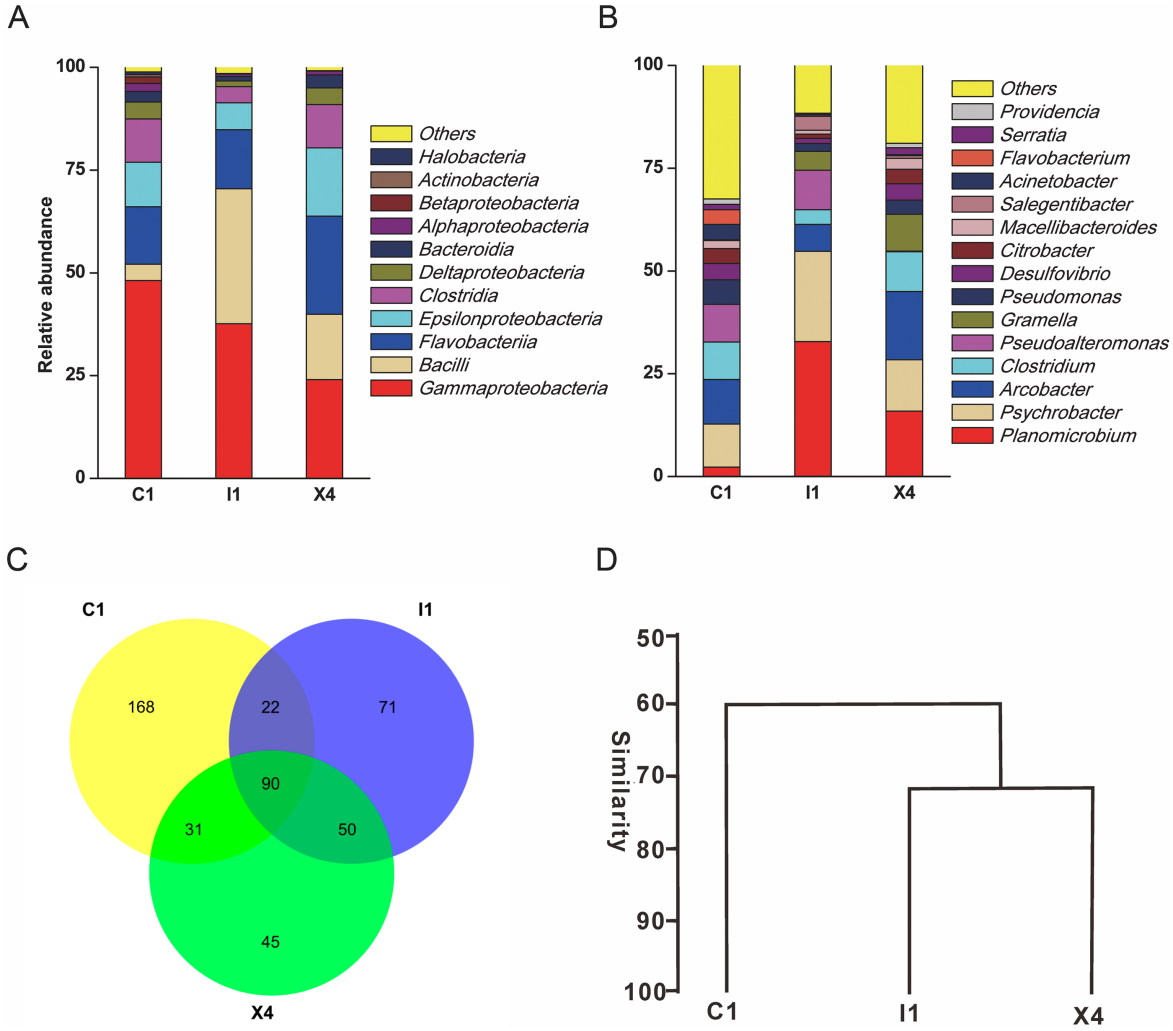
Taxonomic classification of the microbial community at (A) the class level and (B) the genus level. (C) Venn diagram at the OTU level. (D) Hierarchical clustering analysis at the genus level.

A total of 110,628 high-quality partial 16S rRNA sequences were obtained from the three sediment samples by the HTS method. *Gammaproteobacteria* (37%), *Bacilli* (18%), *Flavobacteriia* (17%), *Epsilonproteobacteria* (11%) and *Clostridia* (8%) were the dominant classes in these sediment samples (Fig. 1A). Despite their low relative abundances levels(<1%), *Actinobacteria*, *Alphaproteobacteria*, and *Betaproteobacteria* were present in every sample. *Planomicrobium* (17%), *Psychrobacter* (15%) and *Arcobacter* (11%) were the dominant genera in the sediment samples (Fig. 1B). Cluster analysis results showed that the microbial composition in I1 was more similar to that in X4 than to that in C1 (Fig. 1D). In sediment samples, the range of the ACE was 296-314, while the range of the Chao1 index was 300-314 (Table S2), which are richness indices. C1 produced the largest number of OTUs_(97%)_; correspondingly, the ACE and Chao1 index levels were also highest, followed by those in I1 and X4. C1 contained the most OTUs_(97%)_, followed by I1 and X4, as well as the most unique OTUs_(97%)_ (Fig. 1C).

### The diversity of culturable bacteria detected with different media

**Figure 2:**
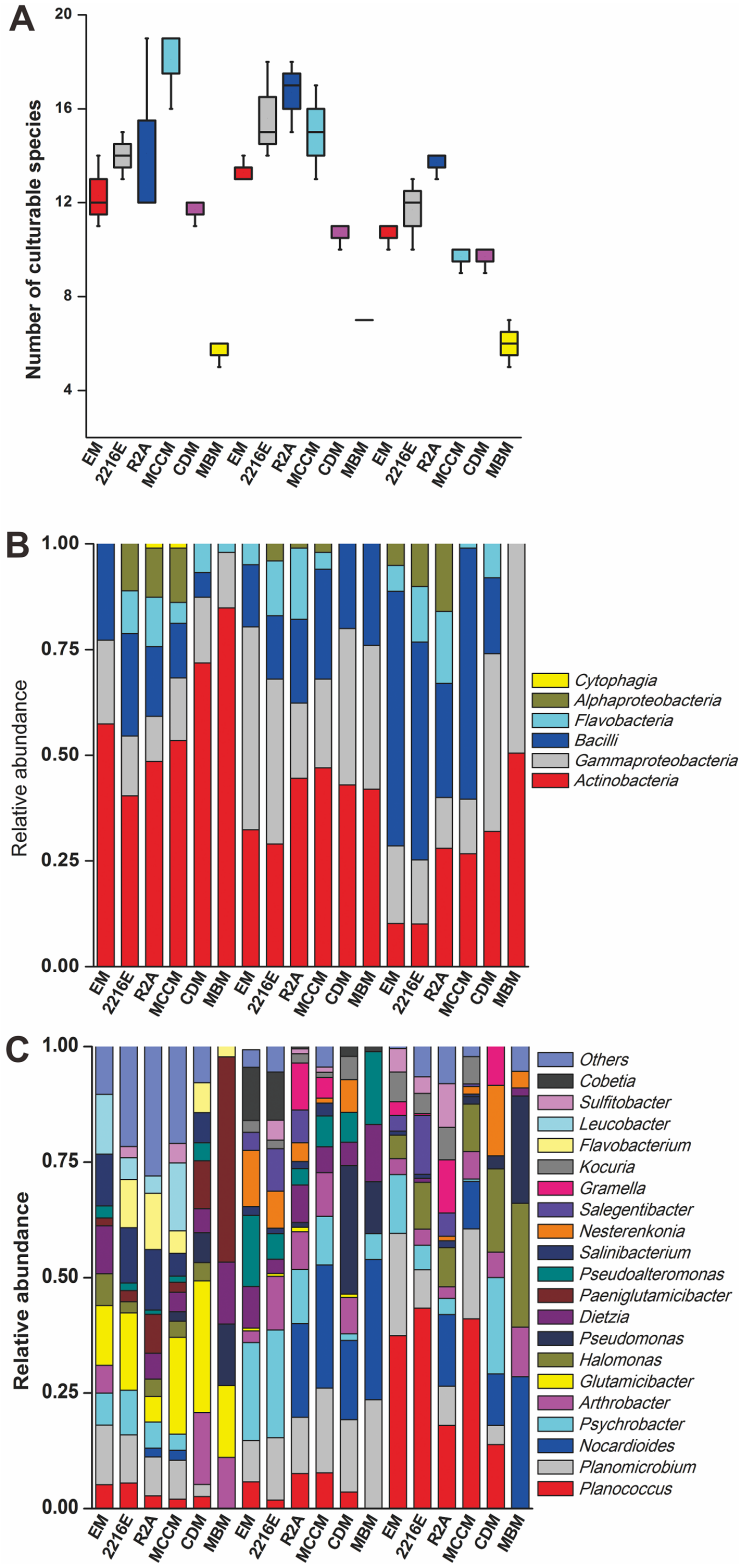
(A) Number of culturable species isolated from C1, I1 and X4 with the six different media. Taxonomic classification at (B) the class level and (C) the genus level.

A total of 594 pure cultures were obtained in this study, including 201 pure cultures from C1, 196 pure cultures from I1, and 188 pure cultures from X4. The culturable bacterial strains were classified into 53 OTUs_(98%)_, 35 genera and 5 classes. At the class level, *Actinobacteria* (10%–84%), *Gammaproteobacteria* (11%–50%) and *Bacilli* (0%–60%) were the main groups determined by the culture method (Fig. 2B). *Cytophagia* (0%–1%) was a rare class, which was isolated only with R2A and MCCM (Fig. 2B). At the genus level, *Glutamicibacter*, *Nocardioides*, *Planococcus*, *Planomicrobium*, *Paeniglutamicibacter*, and *Halomonas* were the main genera. We obtained the most genera from C1 with MCCM. However, we obtained the most genera from I1 and X4 with R2A. Fewer culturable genera were found with CDM and MBM, especially MBM, with which only 16 genera were isolated. Interestingly, we obtained many *Pseudomonas* strains with CDM and MBM, while this genus was not found or was found at low abundance with other media (Fig. 2C). At the OTUs_(98%)_ level, we obtained the most OTUs_(98%)_ with MCCM (40), followed by R2A (37) and 2216E (37). MBM (20) produced the fewest culturable OTUs_(98%)_, followed by CDM (27) and EM (31) (Fig. 2A). No medium resulted in the detection of all 53 culturable OTUs_(98%)_. The total number of culturable OTUs_(98%)_ obtained with these six media was significantly larger than the numbers obtained with single medium (*p* <  0.05) (Fig. S1). In addition, four potentially novel bacterial strains belonging to *Oceaniovalibus*, *Psychrobacter* and *Salegentibacter* were isolated (16S rDNA similarity <  98%) (Kim et al., 2014).

**Figure 3:**
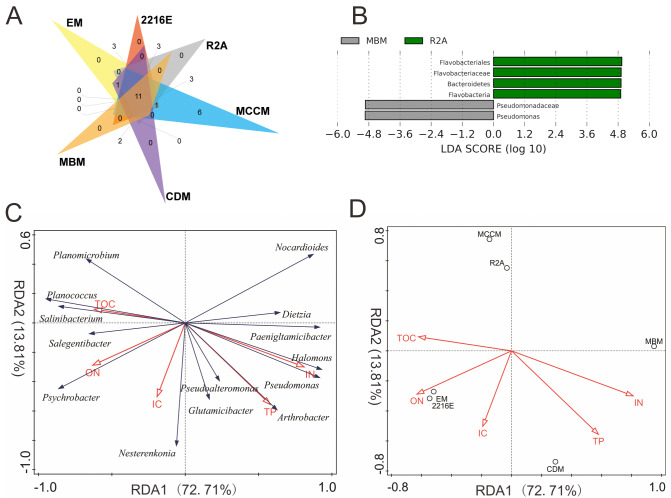
(A) Venn diagram at the species level. (B) LEfSe results at 5 different classification levels. RDA of the relationship between microbial genera (C) and media (D) and physicochemical factors. In LEfSe analysis, yellow nodes represent taxa without intergroup differences, and red and green nodes represent taxa with intergroup differences and high relative abundances in samples.

Among these six media, the shared OTUs_(98%)_ belonged to 8 genera, namely, *Glutamicibacter*, *Planococcus*, *Psychrobacter*, *Pseudoalteromonas*, *Halomonas*, *Dietzia*, *Nesterenkonia*, and *Arthrobacter* (Fig. 3A). MCCM produced the most unique OTUs_(98%)_, such as members of *Pantoea*, *Janibacter*, *Primorskyibacter*, *Devosia* and *Marinomonas*. Interestingly, no unique OTUs_(98%)_ were observed in other media, indicating that MCCM is a special medium for unique bacterial isolation. In addition, the relative abundance levels of *Flavobacteriales*, *Flavobacteria*, *Flavobacteriaceae* and *Bacteroidetes* in R2A and *Pseudomonadaceae* and *Pseudomonas* in MBM were substantially higher than those in other media (Fig. 3B).

### Comparisons of the in situ and cultured microbial diversity

**Figure 4:**
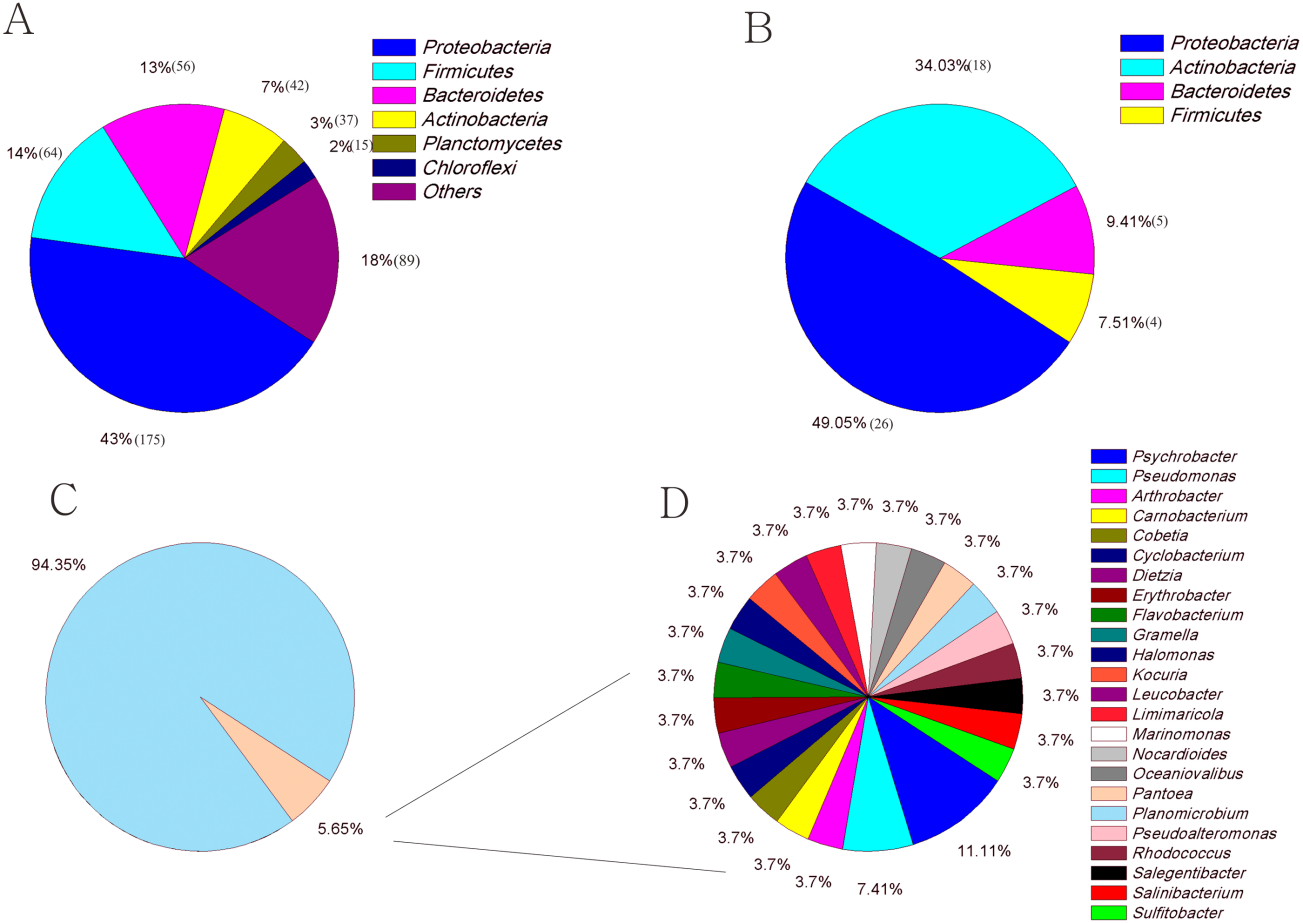
The number of OTUs of each phylum determined with HTS (A) and culture (B) approaches. (C) The fraction of the in situ diversity represented by cultured strains, and taxonomic classification (D). An OTU was considered to be represented by the cultured strains if the OTU had greater than 97% similarity with the 16S rDNA sequence of cultured strains. Taxonomic classification of the OTUs represented by the cultured strains at the genus level.

The culture-dependent and HTS approaches showed different taxonomic distributions at the phylum level, and the number of phyla obtained by the culture-dependent approach was significantly smaller than that obtained by the HTS method (*p* <  0.01) (Fig. S2). Of the 33 phyla in the HTS dataset, only 4 were recovered in the culture collection (Figs. 4A, 4B). A large number of OTUs_(97%)_ in the HTS dataset were assigned to *Proteobacteria* (175), *Firmicutes* (64) and *Bacteroidetes* (56), but the corresponding numbers in the culture collection were much lower (26, 4, and 5, respectively). Among a total of 478 OTUs_(97%)_ from the HTS dataset, 27 were represented by culturable strains, which belonged to 24 different genera, such as *Psychrobacter*, *Pseudomonas* and *Arthrobacter*, accounting for 45% of the total reads in the HTS dataset (Figs. 4C, 4D). Surprisingly, we obtained culturable strains belonging to *Aeromicrobium*, *Janibacter*, *Maribacter*, *Nesterenkonia*, and 3 other genera *that* were not detected by HTS. In the HTS dataset, 58 OTUs_(97%)_ (12% of the total OTUs_(97%)_) had a relative abundance greater than 0.1%, accounting for 95% of the total reads. Among these OTUs(97%), 14 (24%) were shared with the culturable strains (Table S3). These OTUs_(97%)_ were affiliated with the genera *Planomicrobium*, *Psychrobacter*, *Pseudoalteromonas*, *Gramella*, *Pseudomonas*, *Salegentibacter*, *Flavobacterium*, *Arthrobacter*, *Cobetia* and *Halomonas*, which accounted for 45% of the total reads in the HTS dataset (Table S3).

### Physiochemical parameters of the media

The inorganic carbon (IC) concentrations of these six media were between 15.52 mmol/L and 22.33 mmol/L, and no obvious differences were observed. In contrast, a difference in the organic carbon content was identified (Table 2). EM possessed the highest concentration of organic carbon, and the concentration of organic carbon in MBM was the lowest. EM and 2216E were rich in ON, while the amounts of IN in CDM and MBM were greater than the amounts in other media. The nitrogen content in MCCM was surprisingly low. On the basis of these results, EM is a nutrient-rich medium with abundant carbon and nitrogen sources. MCCM is a high-carbon and low-nitrogen medium. CDM and MBM contained a single carbon/nitrogen source.

**Table 2:** Major physicochemical parameters in EM, 2216E, R2A, MCCM, CDM and MBM media. ND means not detected, and values are expressed as the mean ±  standard deviation (SD) (*n* = 3).

Medium	TP (mmol/L)	ON (mmol/L)	IN (mmol/L)	TOC (mmol/L)
EM	6.92 ± 0.17	72.87 ± 2.32	12.31 ± 0.51	1065 ± 12.45
2216E	1.10 ± 0.07	22.68 ± 0.87	7.62 ± 0.39	283 ± 4.32
R2A	1.44 ± 0.06	6.47 ± 0.59	0.56 ± 0.06	161 ± 2.98
MCCM	7.85 ± 0.16	1.80 ± 0.12	0.57 ± 0.04	752 ± 5.34
CDM	27.38 ± 0.82	1.29 ± 0.08	17.33 ± 0.86	229 ± 3.23
MBM	14.12 ± 0.45	ND	52.08 ± 1.06	78 ± 1.45

### Correlations between the culturable bacterial communities and physicochemical factors

The total amount of variation in culturable microbial community composition explained by the first two axes (RDA1 and RDA2) reached 87%. RDA1 was mainly associated with TOC, ON and IN. The IC and TP contents had the largest associations with RDA2 (Fig. 3C). *Salinibacterium*, *Planococcus*, *Salegentibacter*, *Psychrobacter*, and *Planomicrobium* were projected in the negative direction of RDA1; thus, these microbial genera were positively correlated with TOC and ON but negatively correlated with IN and TP. RDA also indicated that only IN contributed significantly to the medium-physicochemical factor relationship (*p* <  0.05), providing 50% of the RDA explanatory power. Moreover, the two media with the greatest IN influence were MBM and CDM, and the medium with the smallest effect was MCCM (Fig. 3D). Although not significant (*p* = 0.052), ON provided 23% of the explanatory power, and 2216E, EM, and CDM were the three most affected media. In addition, the relative abundance levels of *Paeniglutamicibacter*, *Pseudomonas*, and *Halomonas* were positively related to IN (Fig. S3). The relative abundance of *Planomicrobium* was significantly positively correlated with TOC. Furthermore, the relative abundance of *Arthrobacter* increased significantly with increasing TP (Fig. S3).

### Effects of different culture methods on bacterial composition among sediment samples

**Figure 5:**
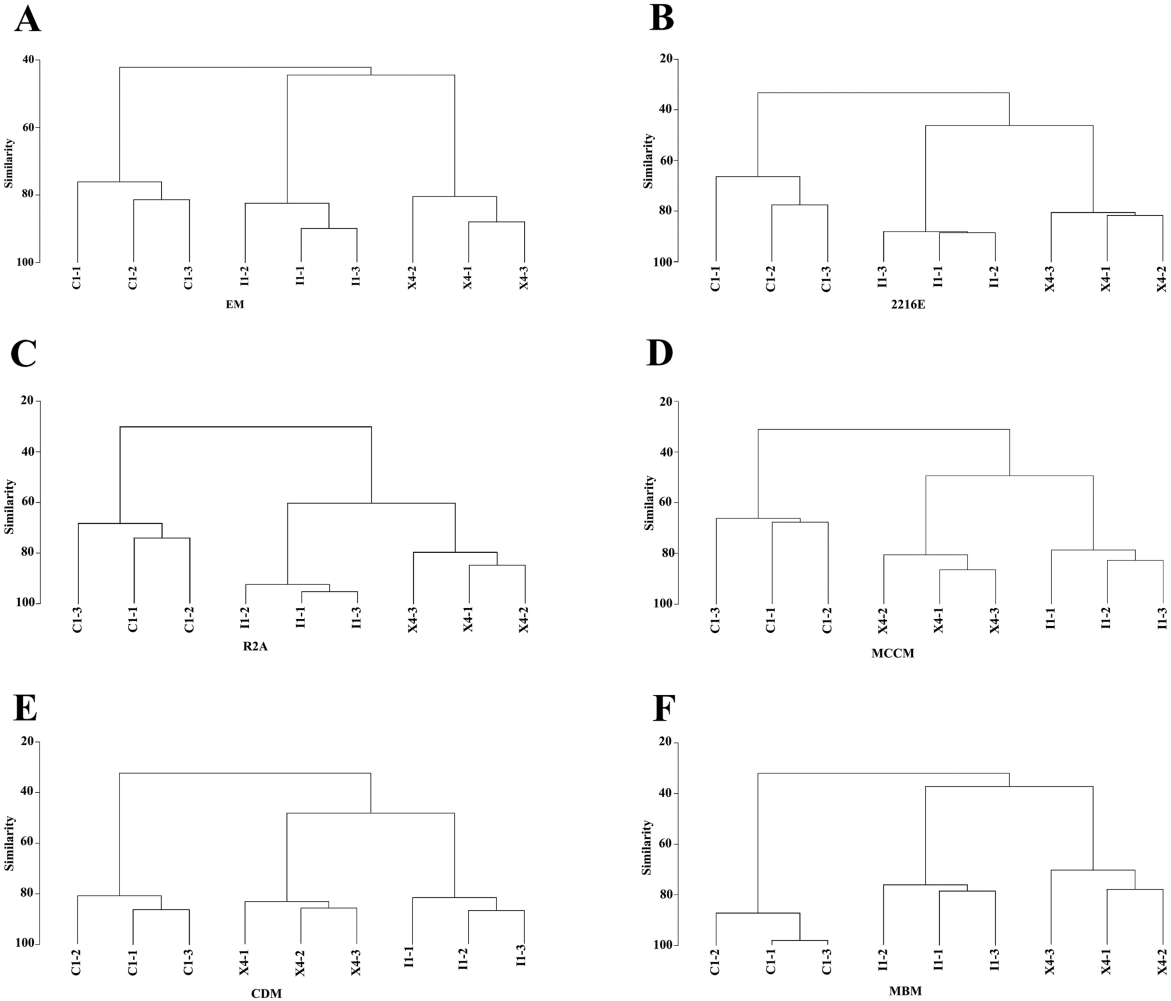
Hierarchical clustering analysis at the genus level to characterize the similarities in culturable bacterial communities among the three sediment samples.

Compared with the culturable bacterial community in C1, that in I1 was more similar to that in X4 (Fig. 5). This result was consistent with that obtained with the HTS method (Fig. 1D). Furthermore, we obtained the largest numbers of culturable OTUs_(98%)_ and unique culturable OTUs_(98%)_ from C1, followed by I1 and X4 (Fig. S4). Similar results were obtained with the HTS method.

## Discussion

Among the total OTUs_(97%)_ from the HTS dataset, only 6% were recovered in the culture collection (Fig. 4C), and high proportions of OTUs_(97%)_ in sediment samples remain uncultured. A multitude of reasons may account for this phenomenon. First, partially nonviable bacteria may be present in sediments (Hahn, Koll & Schmidt, 2019). Manini & Danovaro (2006) found that the dead microbial fraction accounted for an average of 30% of the microbial community in marine sediments. The DNA of these nonviable bacteria does not degrade for a short time; therefore, these nonviable bacteria can be detected by HTS but not by culture method. Second, the applied culture method may be unsuitable for culturing of certain taxa (Hahn, Koll & Schmidt, 2019). Many factors influence the culture of microbes, such as the medium composition, temperature, oxidative stress, and competition or inhibition among bacteria (Stewart, 2012; Tang, 2019). For example, *Clostridia*, a group of strict anaerobes (Wu et al., 2014), dominated libraries generated by HTS from sediment samples (Fig. 1B); however, only aerobic conditions were used in the present study. Any modifications of a single detail may promote the growth of taxa that cannot grow under the original conditions. Tanaka, Nakatsu & Kamagata (2014) revealed that autoclaving of agar in the presence of phosphate, which is a routine procedure in medium preparation, resulted in the formation of H_2_O_2_ and had a negative influence on the diversity of taxa able to grow on the plates In addition, in situ cultivation can recover many previously uncultured bacteria (Kaeberlein, Lewis & Epstein, 2002). The range of factors that may limit microbial culture is vast, complicating identification of specific factors required for or detrimental to cell growth (Tanaka, Nakatsu & Kamagata, 2014). A feasible strategy to overcome this challenge is to use a variety of media and culture conditions. The success of culturomics in the culture of human gut microbiota fully demonstrates the feasibility of this strategy. Lagier et al. (2016) adopted 212 different culture conditions to screen gut bacteria and doubled the number of species isolated from the human microbiome. Third, microbes may be partially able to grow under certain conditions but lack the physiological features necessary to start growing under the provided conditions (Hahn, Koll & Schmidt, 2019). A classic example of the effect of a particular physiological state on bacterial culture is the viable but nonculturable (VBNC) state of living bacteria, which has been widely reported in *Escherichia coli* and *Vibrio*. Such bacteria are able to enter this dormant physiological state if environmental conditions become unsuitable for their growth (Roszak & Colwell, 1987; Oliver, 2010; Hahn, Koll & Schmidt, 2019). In addition, bacterial cells in this state can be stimulated and revived for growth on standard media by specific treatments. For example, supplementation of media with some compounds such as resuscitation-promoting factors is helpful for the revival of bacteria in a VBNC state (Pinto et al., 2011; Li et al., 2014), but the mechanisms of the VBNC state formed and resuscitation remain unclear (Nosho et al., 2018).

Eight bacterial strains belonging to *Janibacter*, *Maribacter*, *Nesterenkonia*, *Devosia*, *Pacificibacter* and *Primorskyibacter* could not be detected by the HTS method but could be isolated by the culture method. On the one hand, the primer bias may be the reason that some cultured taxa were not detected by amplicon sequencing. Intragenomic heterogeneity within 16S rRNA genes may be responsible for the bias in the estimation of prokaryotic diversity. Parada et al. also found that the popular primer pair 515F and 806R would underestimate or overestimate some marine taxa (Sun et al., 2013; Parada, Needham & Fuhrman, 2016). Therefore, due to primer bias, amplicon sequencing may be biased in revealing some environmental microbial groups, and then certain cultured OTUs may not be detected by the amplicon sequencing method. On the other hand, the method of clustering all 16S rRNA sequences occurring within a 97% radius of similarity and then assigning these to “OTUs” from reference trees is widely adopted in 16S rDNA amplicon sequencing. However, this method may reduce phylogenetic resolution, and some sequences below the set threshold cannot be accurately distinguished, which may result in the loss of certain microbes (Callahan et al., 2016). In addition, these strains may also be rare microbes that cannot be detected by molecular technologies (Shade et al., 2012). Due to sequencing depth bias, the culture-independent approach is limited in surveying rare microbes (Hiergeist et al., 2015). These rare microbes may be active and grow rapidly under specific culture conditions, causing them to become the main microbes under suitable conditions (Lynch & Neufeld, 2015; Sun et al., 2018).

Among the six media tested, EM was a nutrient-rich medium. The number of culturable taxa obtained with this medium was small (Fig. 2A, Fig. S1), indicating that nutrient-rich conditions did not increase the diversity of culturable microbes. In nutrient-rich medium, microbes grow rapidly early on and produce a large amount of toxic oxygenic substances such as peroxide, superoxide or hydroxyl radicals. These toxic oxygenic components can cause the death or nonculturability of some microbes (Cho & Giovannoni, 2004). A series of culture experiments also proved that reducing the nutrient composition of medium can improve the diversity of culturable microbes (Cho & Giovannoni, 2004; Dai et al., 2005; Zhang et al., 2010). In addition, this finding to some extent reemphasizes the importance of using compounds known to eliminate free radicals as supplements in microbial media used for isolation of strains from the environment (Stevenson et al., 2004). The addition of free radical scavenging components such as sodium pyruvate and superoxide dismutase to the medium can increase the diversity of culturable microbes (Yue et al., 2006). Tanaka, Nakatsu & Kamagata (2014) also found that autoclaving of agar in the presence of phosphate will result in the formation of H_2_O_2_, causing a significant reduction in the number of CFUs on plates. R2A is an oligotrophic medium containing sodium pyruvate, which can scavenge free radicals, and we obtained the most microbes from this medium (Fig. S1), which also confirms the validity of the above conclusion. The numbers of culturable microbes obtained with CDM and MBM, which contain single carbon and nitrogen sources, were also small (Fig. 2A, Fig. S1). A reasonable explanation for this result is the diversity of microbial nutrient requirements. Köpke also found that the culture approach using various electron acceptors and substrate gradients can improve culturable diversity and argued that a medium containing a wide range of carbon sources would stimulate the growth of different microbes (Köpke et al., 2005).

Nitrogen source provided 73% of the explanatory power in the RDA, and IN significantly affected the composition of culturable microbes (*p* <0.05) (Figs. 3C, 3D). The dominant microbial genera in EM were generally positively correlated with ON, indicating that the bacteria growing in EM may prefer high ON contents. The dominant genera, such as *Planococcus*, *Planomicrobium* and *Pseudoalteromonas,* usually have strong capacities for organic compound degradation and can secrete extracellular proteases, lipases, and other substances to degrade substrates (Chen et al., 2018; Li et al., 2017). Similarly, the relative abundance levels of the dominant microbial genera such as *Pseudomonas* and *Halomonas* in CDM and MBM were usually positively correlated with IN, and these microbes have been proven to have the ability to utilize IN (Ince, Demirbağ & Katı, 2014; Zhong et al., 2015). These microbes can use nitrate reductase, nitrite reductase, glutamine synthetase, and other compounds for nitrogen assimilation (Jetten, 2008; Kanamori, Weiss & Roberts, 1987). The above results suggest that the culturable bacterial communities were related to the components of the media.

## Conclusions

In conclusion, the diversity of microbes obtained with the culture method was substantially lower than that obtained with the HTS method. We found that various media can enhance the number of culturable bacterial taxa detected, and media with rich nutrients or a single type of carbon/nitrogen source are not conducive to the culture of a high diversity of culturable marine bacteria.

##  Supplemental Information

10.7717/peerj.10060/supp-1 Supplemental Information 1 Significant differences in culturable bacteria between the six media combined and single media. * represents *p* < 0.05, ^∗∗^ represents *p* < 0.01

10.7717/peerj.10060/supp-2 Supplemental Information 2 (A) The numbers of phyla and genera revealed by HTS and culture-dependent methods. (B) The fraction of the in situ diversity represented by cultured strains. * represents *p* < 0.05, ** represents *p* < 0.01 OTU-CID: the number of OTUs obtained by amplicon sequencing; OTU-MCD: the number of OTUs with greater than 97% similarity with the 16S rDNA gene of cultured strains. * represents *p* < 0.05, ** represents *p* < 0.01

10.7717/peerj.10060/supp-3 Supplemental Information 3 Relationships between the relative abundances of 14 dominant bacterial genera and physicochemical factors. Adjusted *R*^2^ values and associated *p*-values are shown for each taxonomic group

10.7717/peerj.10060/supp-4 Supplemental Information 4 Venn diagram describing the number of culturable bacteria from different sediment samples

10.7717/peerj.10060/supp-5 Supplemental Information 5 Sampling position. Latitude and longitude of the C1, I1 and X4

10.7717/peerj.10060/supp-6 Supplemental Information 6 Richness and diversity estimates from Illumina libraries of marine sediments from the South China Sea

10.7717/peerj.10060/supp-7 Supplemental Information 7 The OTUs with relative abundances greater than 0.1% in the HTS database. ND means not detected. “Recovered” means that the OTU was represented by a culturable strain

## Additional Information and Declarations

### Field Study Permissions

The following information was supplied relating to field study approvals (i.e., approving body and any reference numbers): The study does not contain any studies with animals, the sampling process without damage to the environment, so official documents and field permits were not required in this study.

### Data Availability

The following information was supplied regarding data availability: The amplicon sequencing data is available at NCBI Sequence Read Archive: PRJNA577514. The 16S rRNA gene sequences of representative species for each sediment sample are available at NCBI GenBank: MK841046 –MK841311.
