# Supplementary figures and images for: Comparison of the diversity of cultured and total bacterial communities in marine sediment using culture-dependent and sequencing methods
Source: PeerJ. 2020 Oct 21;8:e10060. doi: 10.7717/peerj.10060 (PMC7585373; doi:10.7717/peerj.10060)

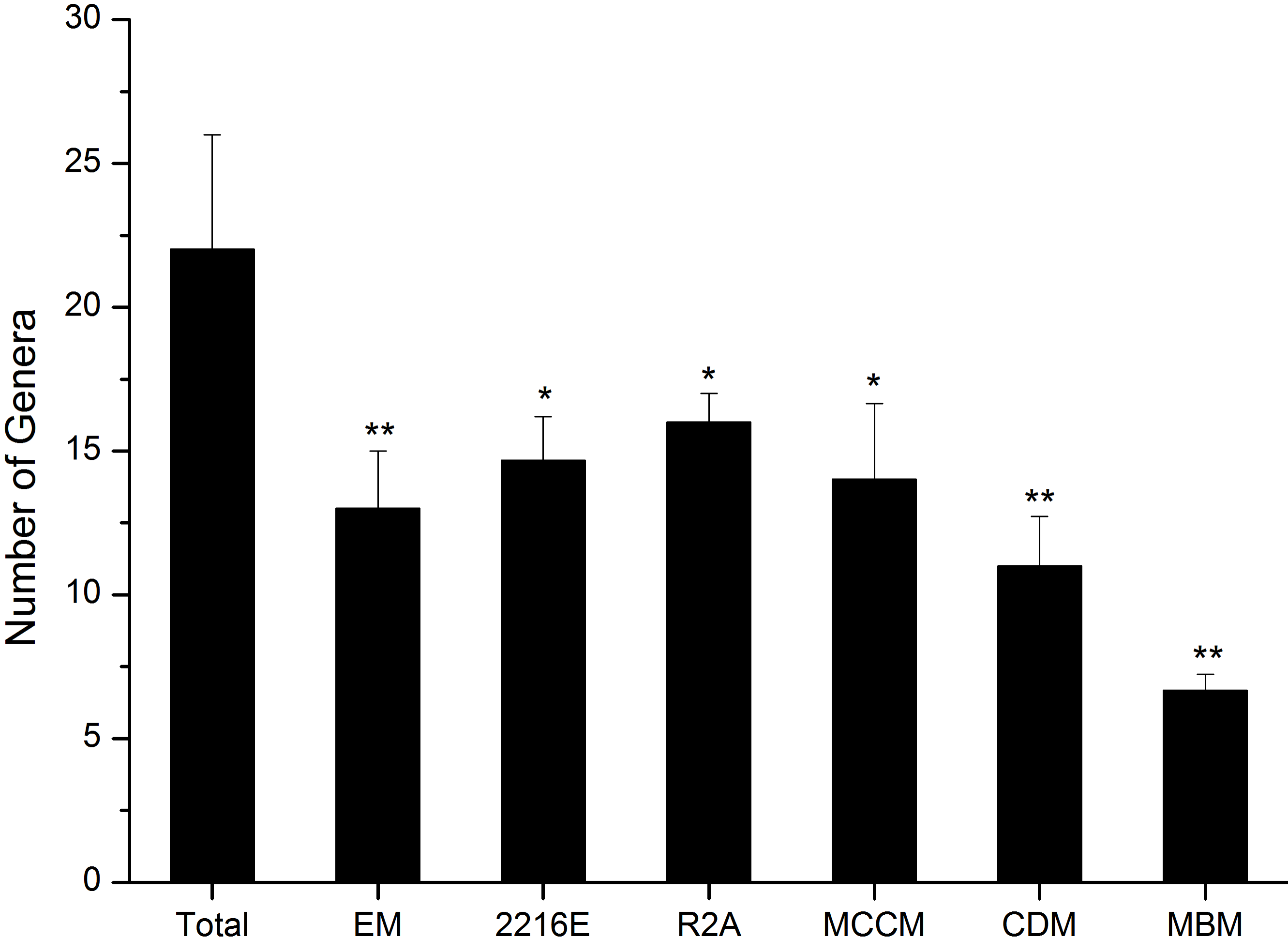

Supplement: Supplemental Information 1 [file peerj-08-10060-s001.png]

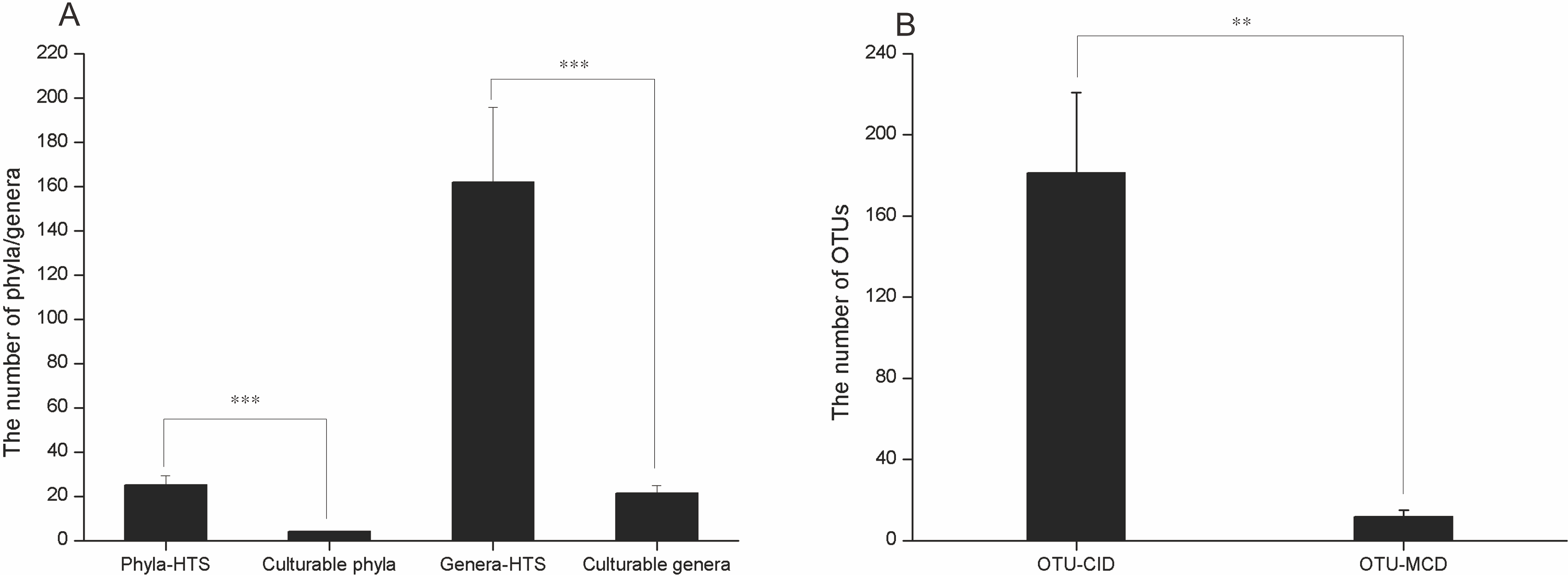

Supplement: Supplemental Information 2 — OTU-CID: the number of OTUs obtained by amplicon sequencing; OTU-MCD: the number of OTUs with greater than 97% similarity with the 16S rDNA gene of cultured strains. * represents p < 0.05, ** represents p < 0.01 [file peerj-08-10060-s002.png]

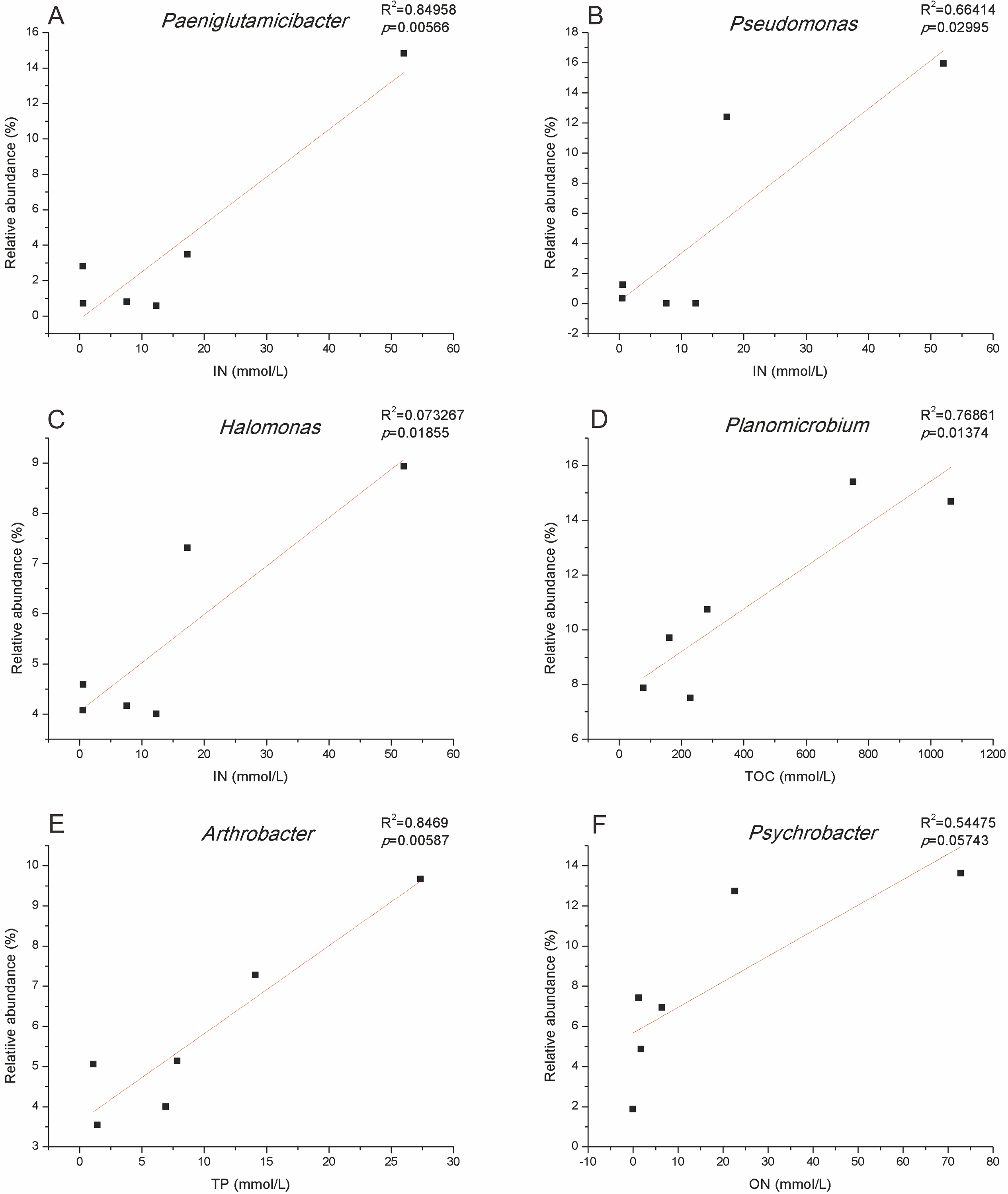

Supplement: Supplemental Information 3 [file peerj-08-10060-s003.png]

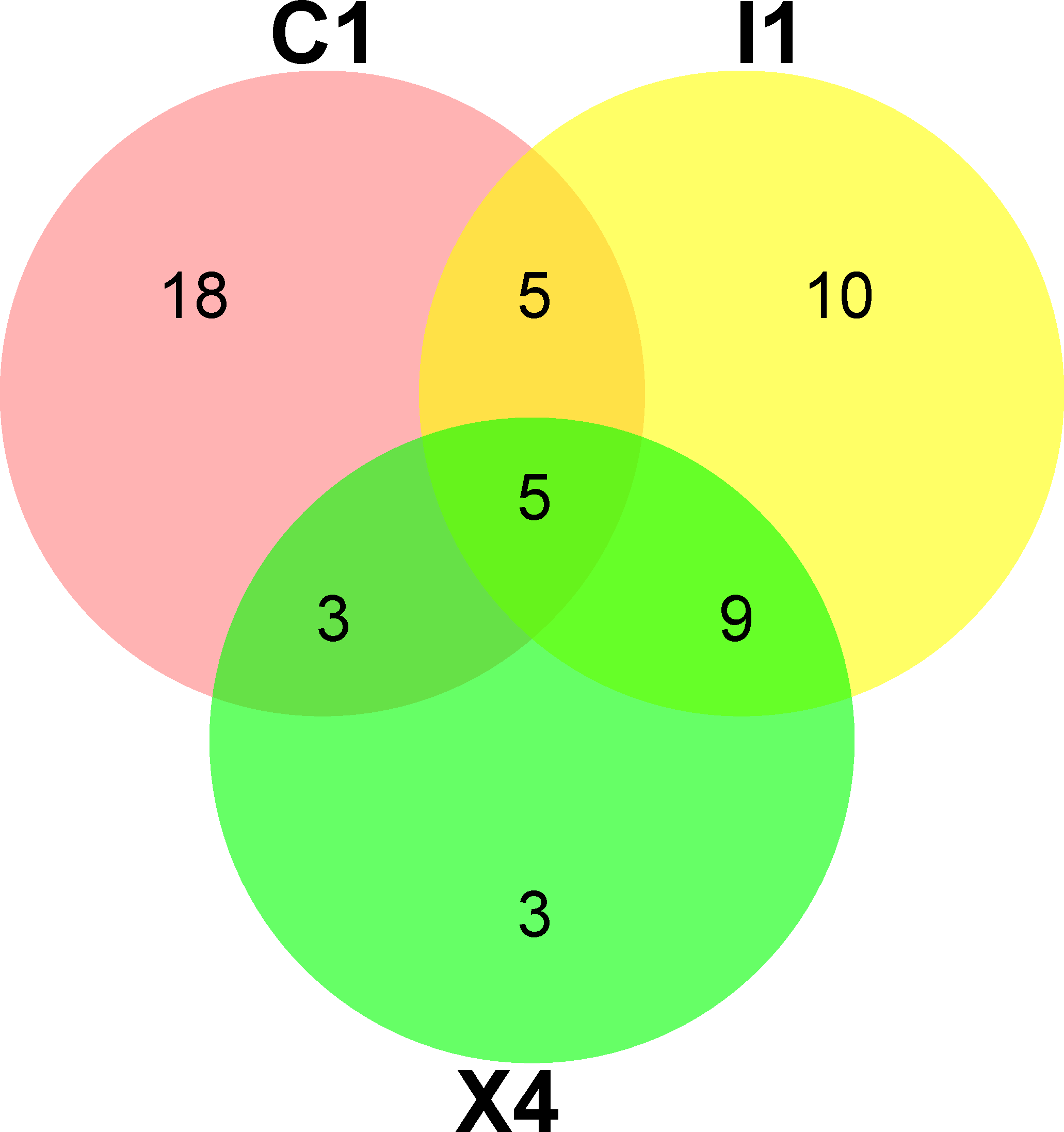

Supplement: Supplemental Information 4 [file peerj-08-10060-s004.png]
