# Supplementary material for: Comparison of the diversity of cultured and total bacterial communities in marine sediment using culture-dependent and sequencing methods
Source: PeerJ. 2020 Oct 21;8:e10060. doi: 10.7717/peerj.10060 (PMC7585373; doi:10.7717/peerj.10060)
Supplement: Supplemental Information 5 [file peerj-08-10060-s005.docx]

**Table S1** Sampling position. Latitude and longitude of the C1, I1 and X4.

| Site | E | N | Depth |
| --- | --- | --- | --- |
| C1 | 117.586° | 21.277° | 630 m |
| I1 | 115.005° | 19.495° | 1556 m |
| X4 | 112.007° | 18.011° | 2446 m |
