# Supplementary material for: Comparison of the diversity of cultured and total bacterial communities in marine sediment using culture-dependent and sequencing methods
Source: PeerJ. 2020 Oct 21;8:e10060. doi: 10.7717/peerj.10060 (PMC7585373; doi:10.7717/peerj.10060)
Supplement: Supplemental Information 6 [file peerj-08-10060-s006.docx]

**Table S2** Richness and diversity estimates from Illumina libraries of marine sediments from the South China Sea.

| Sample | Observed OTUs | Chao1 | ACE | Shannon |
| --- | --- | --- | --- | --- |
| C1 | 312 | 314 | 314 | 3.75 |
| I1 | 234 | 310 | 307 | 2.46 |
| X4 | 217 | 300 | 296 | 2.76 |
