# Supplementary material for: Comparison of the diversity of cultured and total bacterial communities in marine sediment using culture-dependent and sequencing methods
Source: PeerJ. 2020 Oct 21;8:e10060. doi: 10.7717/peerj.10060 (PMC7585373; doi:10.7717/peerj.10060)
Supplement: Supplemental Information 7 [file peerj-08-10060-s007.docx]

**Table S3** The OTUs with relative abundances greater than 0.1% in the HTS database. ND means not detected. “Recovered” means that the OTU was represented by a culturable strain.

| OTU ID | Number of reads | Relative abundance(%) | Phylum | Genus | Recovered |
| --- | --- | --- | --- | --- | --- |
| Otu1 | 17721 | 16.02 | Firmicutes | Planomicrobium | Yes |
| Otu2 | 12523 | 11.32 | Proteobacteria | Arcobacter | No |
| Otu6 | 11061 | 10.00 | Proteobacteria | Psychrobacter | Yes |
| Otu3 | 9412 | 8.51 | Bacteroidetes | NA | No |
| Otu7 | 7052 | 6.37 | Proteobacteria | Pseudoalteromonas | Yes |
| Otu5 | 5328 | 4.82 | Firmicutes | Clostridium | No |
| Otu11 | 4760 | 4.30 | Bacteroidetes | Gramella | Yes |
| Otu9 | 4380 | 3.96 | Proteobacteria | Psychrobacter | Yes |
| Otu13 | 3463 | 3.13 | Proteobacteria | Desulfovibrio | No |
| Otu12 | 3164 | 2.86 | Proteobacteria | Pseudomonas | No |
| Otu18 | 3105 | 2.81 | Proteobacteria | Citrobacter | No |
| Otu17 | 2166 | 1.96 | Firmicutes | Clostridium | No |
| Otu23 | 2058 | 1.86 | Bacteroidetes | Macellibacteroides | No |
| Otu4 | 1540 | 1.39 | Proteobacteria | NA | No |
| Otu26 | 1453 | 1.31 | Bacteroidetes | Salegentibacter | Yes |
| Otu14 | 1378 | 1.25 | Proteobacteria | Acinetobacter | No |
| Otu22 | 1286 | 1.16 | Proteobacteria | Serratia | No |
| Otu20 | 1167 | 1.05 | Bacteroidetes | Flavobacterium | Yes |
| Otu32 | 1033 | 0.93 | Proteobacteria | Providencia | No |
| Otu47 | 874 | 0.79 | Bacteroidetes | Salinimicrobium | No |
| Otu8 | 743 | 0.67 | Proteobacteria | Psychrobacter | No |
| Otu37 | 651 | 0.59 | Proteobacteria | Cobetia | Yes |
| Otu30 | 622 | 0.56 | Firmicutes | Clostridium | No |
| Otu19 | 526 | 0.48 | Proteobacteria | Oceanisphaera | No |
| Otu54 | 495 | 0.45 | Proteobacteria | Stenotrophomonas | No |
| Otu10 | 439 | 0.40 | Bacteroidetes | Bizionia | No |
| Otu21 | 409 | 0.37 | Proteobacteria | NA | No |
| Otu45 | 359 | 0.32 | Proteobacteria | NA | No |
| Otu44 | 356 | 0.32 | Proteobacteria | Oleispira | No |
| Otu121 | 320 | 0.29 | Proteobacteria | Anaerospora | Yes |
| Otu660 | 279 | 0.25 | Bacteroidetes | Mesonia | No |
| Otu63 | 273 | 0.25 | Proteobacteria | Ochrobactrum | No |
| Otu39 | 268 | 0.24 | Firmicutes | Bacillus | No |
| Otu697 | 259 | 0.23 | Proteobacteria | Shewanella | No |
| Otu91 | 257 | 0.23 | Firmicutes | Sedimentibacter | No |
| Otu93 | 251 | 0.23 | Proteobacteria | Pseudomonas | No |
| Otu42 | 249 | 0.23 | Proteobacteria | Pseudomonas | No |
| Otu142 | 245 | 0.22 | Bacteroidetes | Bacteroides | No |
| Otu88 | 235 | 0.21 | Fusobacteria | Fusobacterium | No |
| Otu86 | 233 | 0.21 | Proteobacteria | Pseudomonas | Yes |
| Otu71 | 212 | 0.19 | Proteobacteria | Shewanella | No |
| Otu90 | 212 | 0.19 | Proteobacteria | Pseudomonas | No |
| Otu184 | 197 | 0.18 | Proteobacteria | Pseudomonas | Yes |
| Otu85 | 196 | 0.18 | Proteobacteria | Acinetobacter | No |
| Otu40 | 191 | 0.17 | Proteobacteria | NA | No |
| Otu61 | 183 | 0.17 | Proteobacteria | Escherichia | Yes |
| Otu73 | 177 | 0.16 | Proteobacteria | Cupriavidus | No |
| Otu166 | 169 | 0.15 | Chloroflexi | NA | No |
| Otu53 | 161 | 0.15 | Proteobacteria | Comamonas | No |
| Otu144 | 151 | 0.14 | Proteobacteria | Halomonas | Yes |
| Otu112 | 141 | 0.13 | Firmicutes | Oscillospira | No |
| Otu138 | 132 | 0.12 | Proteobacteria | Paracoccus | No |
| Otu25 | 132 | 0.12 | Bacteroidetes | Flavobacterium | No |
| Otu77 | 132 | 0.12 | Proteobacteria | NA | No |
| Otu41 | 128 | 0.12 | Proteobacteria | NA | No |
| Otu106 | 117 | 0.11 | Actinobacteria | Arthrobacter | Yes |
| Otu111 | 117 | 0.11 | Proteobacteria | Mycoplana | No |
| Otu43 | 112 | 0.10 | Proteobacteria | Acidovorax | No |
